# Arterial Pulse Tapping Artifact Mimicking Acute Coronary Syndrome on Electrocardiogram

**DOI:** 10.1016/j.jaccas.2026.107945

**Published:** 2026-05-20

**Authors:** Mriganka Nerkar, Katharine Rainer, Jesse Frye, David T. Zhang, Mark B. Hellerman

**Affiliations:** a Renaissance School of Medicine at Stony Brook University, Stony Brook, New York, USA; b Division of Cardiology, Department of Medicine, Weill Cornell Medical Center, New York, New York, USA; c Division of Cardiology, Department of Medicine, Stony Brook University Hospital, Stony Brook, New York, USA

**Keywords:** arterial pulse tapping artifact, Aslanger's sign

## Abstract

**Introduction:**

Arterial pulse tapping artifact is a deceptive electrocardiographic artifact caused by mechanical movement of an electrode from arterial pulsation, which can produce pseudo-ischemic ST-T abnormalities.

**Case Presentation:**

An 88-year-old woman presented with substernal chest discomfort that was burning in quality and radiated to her back. Vital signs were stable. Physical examination was benign.

**Discussion:**

The presence of bizarre, nonphysiologic ST-T wave distortion, which spares a single limb lead, allowed for prompt recognition of arterial pulse tapping artifact. Recognizing this pattern—and confirmation with electrode repositioning—prevented misdiagnosis of acute coronary syndrome and inappropriate invasive angiography.

## Graphical Abstract

**Figure:**
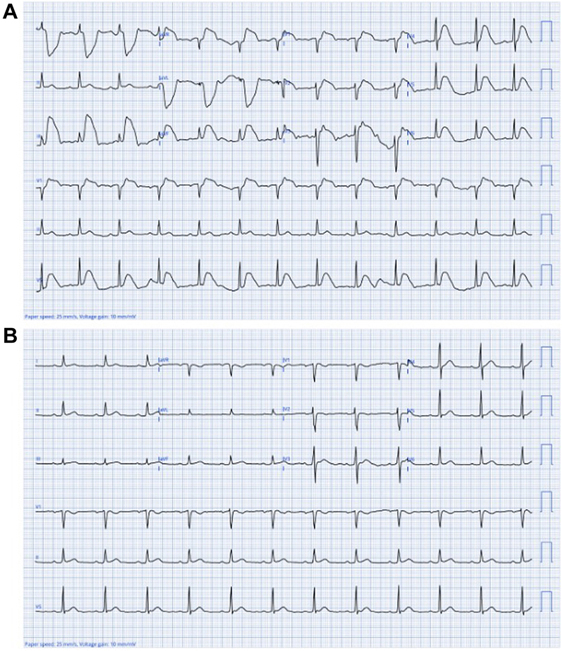

## History of Presentation

**Figure 1:**
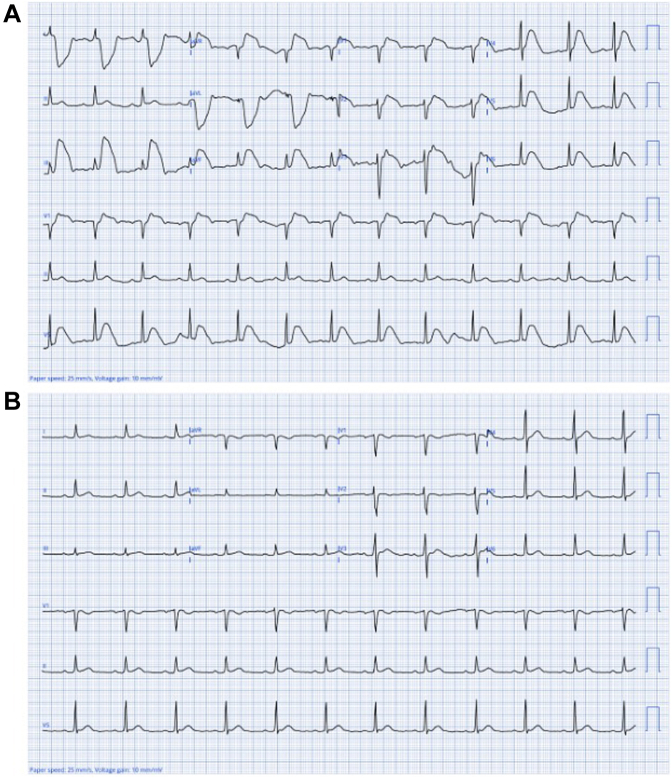
Initial and Repeat Electrocardiograms Electrocardiogram (A) before and (B) after electrode repositioning.

An 88-year-old woman presented to the emergency department for chest pain that began 2 hours prior to arrival. She described the pain as substernal, burning in quality, with radiation to the back. The pain was nonpleuritic, not associated with exertion, and started shortly after her last meal. Medical history was notable for anxiety and depression. Vital signs were stable. Cardiovascular examination was benign. Her initial electrocardiogram (ECG) is shown in Figure 1A.

**Take-Home Message**

• Arterial pulse tapping artifact can be recognized by the presence of bizarre ST-T wave distortion sparing a single limb lead and can be quickly confirmed with electrode repositioning.

Question: What is the most likely diagnosis based on the patient's initial ECG?

A. Brugada pattern
B. Acute occlusion of a coronary vessel
C. Arterial pulse tapping artifact
D. Sodium channel blocker toxicity

Choice A is incorrect. Brugada pattern is characterized by coved ST-segment elevation in V_1_ and V_2_ with a high takeoff ≥2 mm at the end of the QRS, followed by a symmetric negative T-wave. This ECG does not demonstrate true ST-segment elevation but rather a bizarre T-wave that occurs at a fixed interval of roughly 40 milliseconds after the QRS complex.

Choice B is incorrect. The ST-segment elevation associated with the acute occlusion of a coronary vessel tends to be confined to contiguous leads corresponding to anatomic territories. It is often associated with reciprocal ST-segment depression in electrically opposite leads. The illusion of ST-segment elevations and depressions in this ECG does not correspond to a single coronary distribution.

Choice C is correct. This ECG demonstrates bizarre, nonphysiologic ST-T wave distortion, sparing a single limb lead. Among the augmented leads, aVL is most distorted, allowing localization of the culprit extremity to the left arm.

Choice D is incorrect. Sodium channel blocker toxicity is characterized by intraventricular conduction delay with QRS widening—particularly in lead II—associated with a prominent R′ wave in aVR and often sinus tachycardia. The QRS complex in this ECG is narrow, without a true R′ in aVR or sinus tachycardia. In addition, the apparent ST-segment abnormality occurs after a brief return of the tracing to baseline after the QRS complex. In true pathologic ST-segment elevation—such as that seen with Brugada pattern, myocardial infarction, or sodium channel blocker toxicity—the ST segment begins directly at the J-point without an intervening return to baseline.

## Discussion and Rationale

Arterial pulse tapping artifact can closely mimic ischemic ST-segment changes, but it is a mechanical artifact caused by arterial pulsation transmitted to an ECG electrode overlying a pulsatile vessel.^1^^,^^2^

Applying the principles of Einthoven's triangle, the presence of equal-amplitude artifact in leads I and III—without involvement of lead II—localizes the source of artifact to the left arm electrode (Video 1). Consistent with this, and corroborating the left arm as the culprit electrode, augmented leads aVR and aVF display artifact amplitudes approximately half that seen in aVL.^3^

The presence of bizarre ST-T wave distortion not corresponding to a coronary distribution, which spares a single limb lead, allowed for prompt recognition of the artifact and ultimately prevented unnecessary invasive management. A repeat ECG after repositioning the left arm electrode is shown in Figure 1B. The patient's pain resolved after initiation of a proton pump inhibitor and was ultimately attributed to gastroesophageal reflux.

## Funding Support and Author Disclosures

The authors have reported that they have no relationships relevant to the contents of this paper to disclose.

## Appendix

Video 1 Electrocardiographic Localization of Arterial Pulse Tapping Artifact Animated schematic demonstrating how arterial pulse tapping artifact affecting the left arm (LA) electrode produces pseudo–ST-T wave abnormalities, with equal artifact in leads I and III, sparing lead II, and maximal distortion in aVL. LL = left leg; RA = right arm.
